# Rapid nondestructive detection of peanut varieties and peanut mildew based on hyperspectral imaging and stacked machine learning models

**DOI:** 10.3389/fpls.2022.1047479

**Published:** 2022-11-10

**Authors:** Qingsong Wu, Lijia Xu, Zhiyong Zou, Jian Wang, Qifeng Zeng, Qianlong Wang, Jiangbo Zhen, Yuchao Wang, Yongpeng Zhao, Man Zhou

**Affiliations:** ^1^ College of Mechanical and Electrical Engineering, Sichuan Agricultural University, Yaan, China; ^2^ College of Food Sciences, Sichuan Agricultural University, Yaan, China

**Keywords:** peanut seeds, variety classification, mildew detection, stacked ensemble learning model, nondestructive testing

## Abstract

Moldy peanut seeds are damaged by mold, which seriously affects the germination rate of peanut seeds. At the same time, the quality and variety purity of peanut seeds profoundly affect the final yield of peanuts and the economic benefits of farmers. In this study, hyperspectral imaging technology was used to achieve variety classification and mold detection of peanut seeds. In addition, this paper proposed to use median filtering (MF) to preprocess hyperspectral data, use four variable selection methods to obtain characteristic wavelengths, and ensemble learning models (SEL) as a stable classification model. This paper compared the model performance of SEL and extreme gradient boosting algorithm (XGBoost), light gradient boosting algorithm (LightGBM), and type boosting algorithm (CatBoost). The results showed that the MF-LightGBM-SEL model based on hyperspectral data achieves the best performance. Its prediction accuracy on the data training and data testing reach 98.63% and 98.03%, respectively, and the modeling time was only 0.37s, which proved that the potential of the model to be used in practice. The approach of SEL combined with hyperspectral imaging techniques facilitates the development of a real-time detection system. It could perform fast and non-destructive high-precision classification of peanut seed varieties and moldy peanuts, which was of great significance for improving crop yields.

## 1 Introduction

Peanuts are considered as important edible oil raw materials and national economic food crops (Wang et al., 2021a). In recent years, in order to meet the growing demands of agriculture and industry, seed hybridization technology has been widely used, so that the number of peanut seed varieties has increased significantly. However, there are many processes that can lead to varietal intermingling throughout growth and development, such as planting, harvesting, transport and storage. At the same time, different varieties of peanuts adapt to different soil types, climatic environments and cultivation methods. Therefore, it is particularly important to identify the purity of peanut seeds before sowing (Liu et al., 2022). In addition, moldy peanut seeds are severely damaged by mold, and the nutrients of the seeds are destroyed in a large amount, resulting in seed rot and weak seedlings, thereby reducing seed vigor and yield (Pasupuleti et al., 2016; Sharma et al., 2021). The purity of peanut seed varieties and the quality of peanuts have a profound impact on the final yield of peanuts and the economic benefits of farmers. Therefore, it is of great value to identify the variety and quality of peanut seeds before sowing.

Peanut seeds of different varieties have very similar appearance properties. The traditional identification method is to identify the shape, skin and color of peanuts manually, but this methods have the disadvantages of low analysis efficiency and time-consuming and labor-intensive (Yuan et al., 2020). At the same time, improper storage of peanuts is prone to produce aflatoxin, which will seriously affect the germination rate of peanut seeds (Sharma et al., 2021). Quantitative measurement methods such as thin-layer chromatography, gas chromatography, and high-performance liquid chromatography are widely used for the determination of aflatoxin content due to their high sensitivity (Wang et al., 2014). However, these ways are destructive, time-consuming, complex to operate, and difficult to implement online. In order to overcome the drawbacks brought by traditional detection methods, rapid and non-destructive detection techniques, such as Raman spectroscopy (Kopec and Abramczyk, 2022), machine vision (Mohi-Alden et al., 2022), and near-infrared spectroscopy (Jiang et al., 2022), have been applied to the classification of agricultural products. When detected by Raman spectroscopy, organic molecules can easily convert the absorbed photons into fluorescent molecules and produce fluorescence effects. Its intensity is much higher than that of the Raman spectral peak, and it can even completely cover the entire Raman spectrum. In this case, surface-enhanced Raman spectroscopy is required (Pang et al., 2020; Xu et al., 2020). Machine vision technology has been used for peanut loss detection. While machine vision technology is commonly used to evaluate the appearance attributes of peanuts, it cannot evaluate internal quality attributes. Near-infrared spectroscopy is a mature non-destructive testing technology that can be used for non-destructive testing of complex samples (Zhang et al., 2022). However, the composition of some samples (eg food) is often heterogeneous. If the spatial distribution of its components is not considered, a large amount of important information may be lost, affecting subsequent analysis results (Badaró et al., 2021).

Hyperspectral imaging (HSI) technology can simultaneously obtain spectral information and spatial position information of the sample (Liu et al., 2019; Su et al., 2021). It has been proven to be a fast, non-invasive and effective tool for food quality analysis (Cortés et al., 2019). Recently, HSI has been used for food classification, ingredient detection, agricultural product quality detection, and damage detection, etc (Xiang et al., 2022). HSI is characterized by multiple bands and high spectral resolution (Tan et al., 2018). (Jin et al., 2022) used the spatial spectral features of HSI to classify peanut seeds, and the classification accuracy reached 97.64%. (Qi et al., 2019) used HSI technology and joint sparse representation model to identify fungi contaminated peanuts. (Sun et al., 2020) used HSI technology combined with chemometrics to detect the fat content in peanut kernel. These studies reveal the potential of HSI in peanut detection, but further research is still necessary.

Classification using HSI is usually achieved by machine learning methods, such as traditional methods such as support vector machines and random forests. However, traditional machine learning has low computational efficiency and accuracy for HIS with large data volumes. SEL improves predictive potential and adjusts the bias-variance trade-off of machine learning submodels. The stacking strategy is an approach based on the “wisdom of the crowd”, which maximizes the generalization accuracy by employing the base learning model to form an ensemble model (Zandi et al., 2022). In theory, different base learners can give full play to the cooperative advantages of ensemble learning and achieve the effect of complementary integration (He et al., 2022). Stacked ensemble machine learning algorithms have been successfully used in various applications including wind power prediction (Dong et al., 2021), soil classification (Eyo and Abbey, 2022), species classification (Fu et al., 2022), etc. (Zhang et al., 2020) classifies vegetation based on medium resolution spectral imaging technology and SEL, and its accuracy is 5.1-5.2% higher than other single models. (Fu et al., 2022) constructed a model based on multispectral images and SEL, and found that the integrated learning algorithm produced better classification performance than the basic model, with an overall accuracy rate of 1.6-12.7% higher. There are relatively few studies using SEL for fine classification of peanut seed varieties and mildewed peanuts, and there is a lack of comparative research on the classification ability of the SEL algorithm for peanut seeds using the HSI.

In this study, a method of HIS combined with SEL was proposed, and the characteristic wavelength was used to realize the classification of peanut seeds and the identification of mildew in peanut seeds. This article aims to: 1) Develop a method based on HIS combined with SEL to realize variety classification and mildew detection of peanut seeds. 2) Explore the influence of the feature wavelengths selected by different variable selection methods on the classification model to determine the best features. 3) Establish a stacked ensemble model with high classification accuracy for peanut seed variety and peanut seed mildew. 4) Evaluate and compare the classification performance of the base model and the stacked ensemble model on samples.

## 2 Materials and methods

### 2.1 Sample preparation

This study involved four main peanut varieties in key cultivation areas in my country (Shandong, Henan, Jiangsu, etc.), including Dabaisha, Huayu, Xiaobaisha, and Luhua. All peanut seeds were sourced from a Chinese commercial seed company and picked at random. There were 400 grains of each peanut variety, all samples were normal, and the appearance was clean and complete. To obtain naturally moldy peanuts, the peanuts were placed in a constant temperature and humidity incubator. The optimum temperature and relative humidity for Aspergillus flavus growth and aflatoxin production are 37°C and 90%, and 28°C and 90%, respectively. (Lattab et al., 2012; Yuan et al., 2020).Therefore, the peanuts were placed in the incubator first at 37°C and 90% humidity for 10 days to facilitate the rapid development and reproduction of Aspergillus flavus. From the 11th day, the temperature of the constant temperature and humidity incubator was set to 28°C, and the relative humidity remained unchanged. Then, on the 20th and 30th days, some peanuts with the same degree of mildew were taken out as moldy peanut samples. In order to verify whether peanuts contain aflatoxin, after obtaining HSI, the AFB1 rapid detection card produced by Shenzhen Fender Technology Co., Ltd. was used to detect the residues of AFB1 in various varieties of peanuts. Peanut samples of each variety were tested, and 150 moldy peanut seed samples were selected for each variety from the peanut seeds detected as moldy.

### 2.2 Hyperspectral imaging system

**Figure 1:**
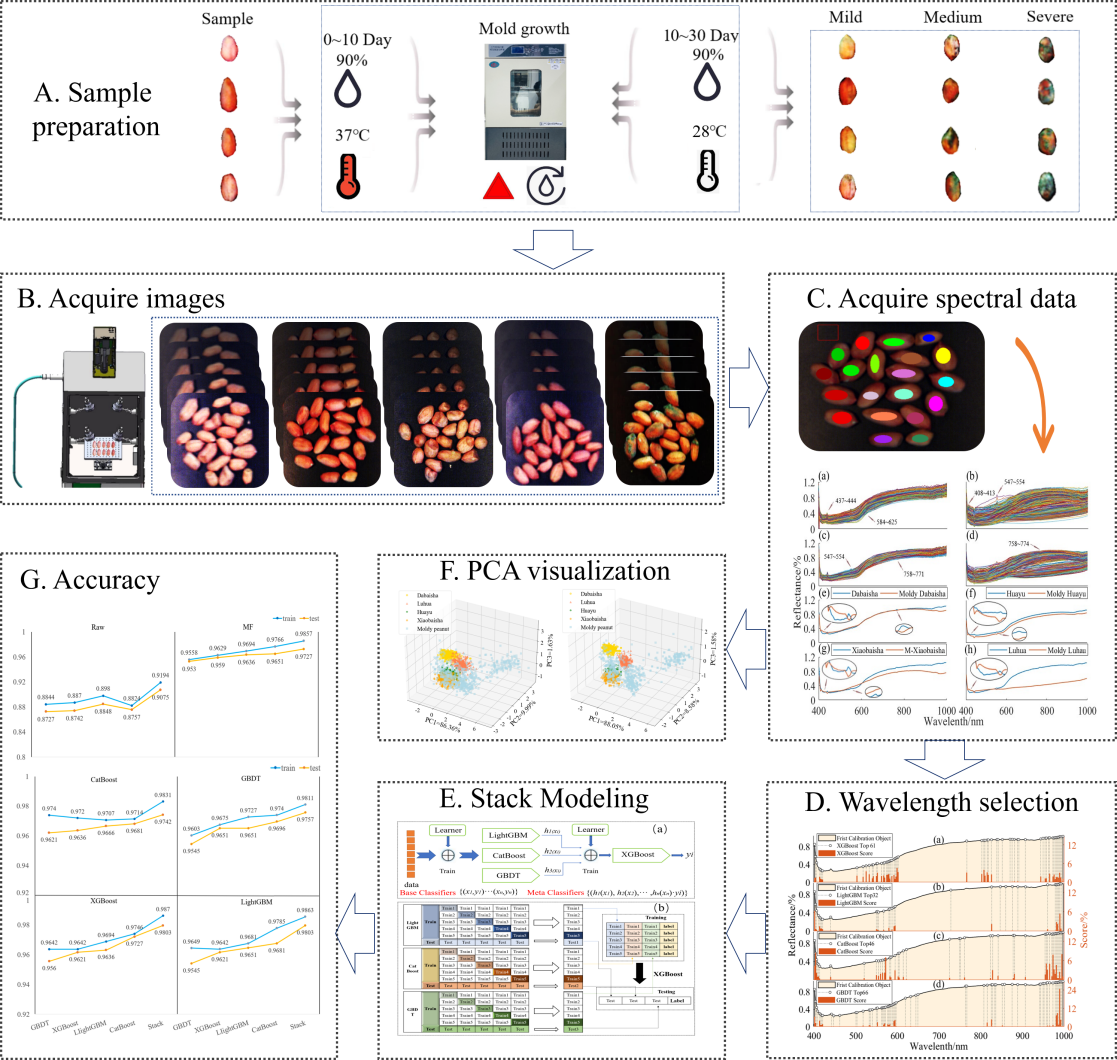
Flowchart of the main steps in the detection of peanut seeds by hyperspectral imaging technology.

The hyperspectral image acquisition system adopts the Image-λ “spectral image” series hyperspectral machine of Zhuolihanguang Company, and uses SpacVIEW software to operate it. The system consists of a computer, a transmission platform, a dot matrix camera and a halogen light source. As shown in **Figure 1**, the effective band range of its spectrum is 400-1000nm, the band resolution is 2.8nm, a total of 235 bands, and the pixel is 1344*1024. The measured display properties R, G and B of each group of samples were set to 638.7, 551.58 and 442.95 respectively, the time was set to 10s, the distance between the peanut sample and the camera lens was set to 165mm, and the moving speed of the sample was set to 4.7mm·s^-1^. The exposure time of the camera was 4ms, and the scanning area of the spectrum was 150mm. The hyperspectral camera and the sample peanut belong to the vertical scanning relationship. The sample is placed on the transmission platform, and the hyperspectral camera is perpendicular to the transport platform. The hyperspectral image is collected through the uniform movement of the transmission platform.

### 2.3 Hyperspectral image acquisition and correction

**Figure 2:**
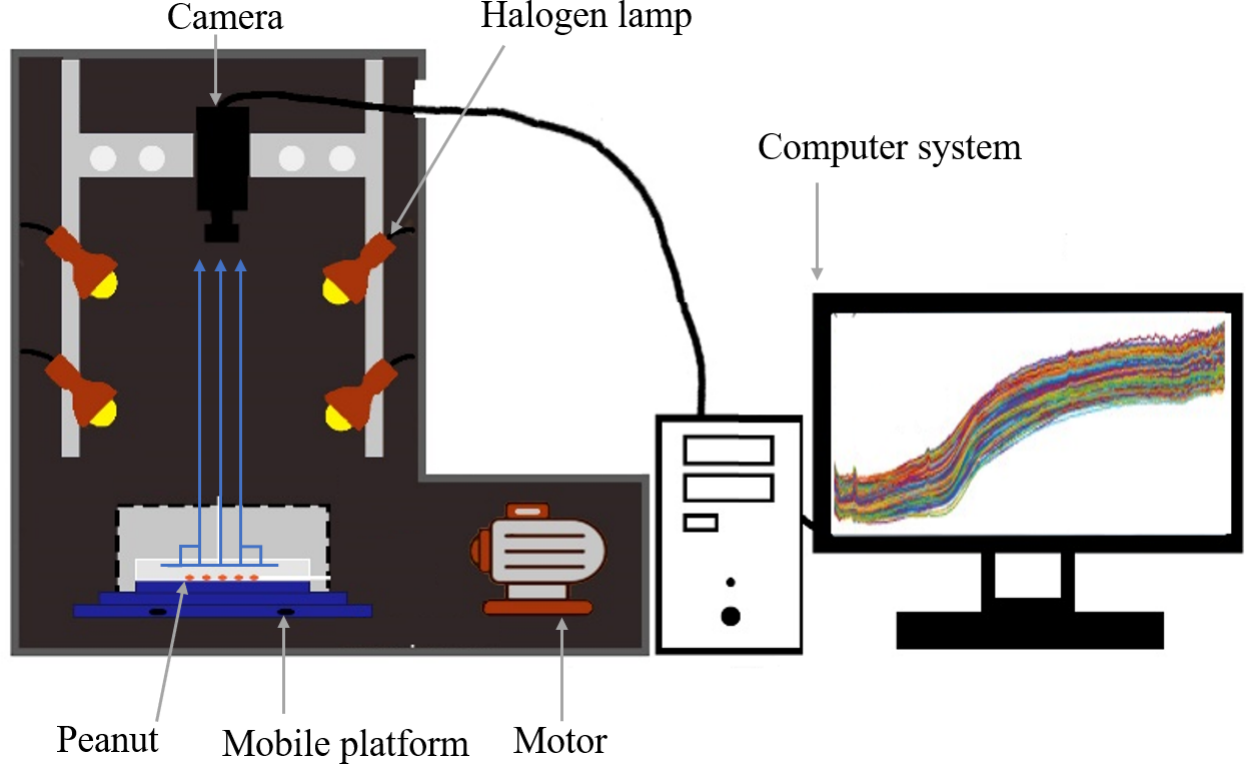
Hyperspectral imaging system.

Image processing was first performed to identify regions of interest (ROI), each defined as the inner contour region of a peanut seed. During the acquisition process, due to the influence of noise caused by the surrounding environmental factors and the dark current of the instrument, it is necessary to collect the black frame and white frame of the image separately before collecting the sample. After the sample collection was completed, the black and white correction was performed in SpacVIEW according to the following formula (1.1), and the ENVI5.1 software was used to extract the area required for the experiment in the peanut hyperspectral image after black and white correction. Then, the average reflectance value of the spectral data on the extracted area was calculated as the characteristic reflectance spectral curve of different varieties of peanuts, as shown in **Figure 2**. When collecting the spectral data of the sample, the sample to be tested is affected by illumination, dark current, light scattering and human operation when taking pictures, resulting in noise and a large amount of interference information in the spectral data (Li et al., 2021). The raw visible-NIR spectra also require baseline correction and noise removal. Therefore, all these unwanted components must be removed to improve the signal-to-noise ratio and optimize model performance. In order to obtain more valuable spectral data, this paper used Median Filtering (MF) to preprocess the spectral data. MF has a good filtering effect on impulse noise, especially while filtering out noise, it can protect the edge of the signal so as not to be blurred, and obtain more valuable spectral data (Kumar et al., 2021).


(1.1) $$R_{1}=\frac{R_{0}-R_{B}}{R_{w}-R_{B}}$$

In the formula, *R*_0_ is the initial hyperspectral image (RAW), *R*_1_ is the image after black and white correction, *R*_*B*_ is the black frame of the image collected after closing the lens, and *R*_*w*_ is the white frame of the image collected after the acquisition and debugging are correct.

### 2.4 PCA spectral data visualization

Principal Component Analysis (PCA) was proposed by Pearson in 1901 and later popularized by Hotelling in 1933. The main idea of PCA is to map m-dimensional features to k-dimensions (k<m), and linearly combine many original feature factors with certain correlation into several new independent comprehensive factors. And, as much as possible to reflect the original information of these characteristic factors (Bianchi et al., 2015). A 3D scatter plot of the first three principal components was used to visualize the separation of peanut seed samples.

### 2.5 Feature wavelength selection

The raw spectral data collected by the hyperspectral system is affected by a large amount of redundant information, resulting in a decrease in classification accuracy (Zou et al., 2023). The raw spectral data collected by hyperspectral systems are composed of a large number of bands and have multicollinearity. It is advisable to select some important variables to develop more powerful and concise classification models. Characteristic wavelength modeling can effectively eliminate the redundancy of spectral data and improve the accuracy and efficiency of classification models. Therefore, it is necessary to extract the characteristic wavelengths with strong correlation to judge the type of samples from the original spectral data. The spectral data of peanut seed samples contains 235 characteristic bands. This paper adopted four effective wavelength extraction methods: XGBoost, LightGBM, GBDT, and CatBoost. The contribution rate of each wavelength was obtained through cross-validation, and the wavelength with high contribution rate was selected, thereby simplifying the establishment of subsequent models and reducing the amount of calculation.

### 2.6 Classification model

Based on the above-mentioned spectral preprocessing and effective feature wavelength selection methods, five machine learning algorithms, GBDT, XGBoost, CatBoost, LightGBM and SEL, were used to establish classification models. Determine the optimal model based on the prediction results. 70% of the spectral data was randomly selected as the training set, and the remaining 30% of the spectral data was used as the test set. The samples were divided into 5 categories, including Dabaisha, Huayu, Xiaobaisha, Luhua, and moldy peanut seeds.

XGBoost is an optimized distributed gradient boosting library designed to be efficient, flexible and portable (Li et al., 2022). First, it constructs an appropriate number of weak learners, mainly classification regression trees, to train weak learners. It also performs weighting calculations and summations after training to get the final classification model. XGBoost uses a second-order Taylor expansion for the loss function. At the same time, XGBoost also supports column sampling to avoid overfitting and reduce the computational workload. After each iteration, XGBoost assigns the learning rate to leaf nodes, reducing the weight of each tree and providing better space for subsequent learning (Liu et al., 2021b).

LightGBM was originally developed by researchers at Microsoft and Peking University to address the efficiency and scalability issues of GBDT and XGBoost when applied to high-dimensional input features and large data volumes (Wen et al., 2021). The core concepts of LightGBM are histogram algorithm, leaf growth strategy with depth limit, support category features, histogram feature optimization, multi-threading optimization and cache hit ratio optimization (Wang et al., 2021b). The algorithm bins the original continuous feature values and uses these bins to build a model. The histogram greatly reduces the time consumption of split point selection and improves the training and prediction efficiency of the model (Liu et al., 2021a).

CatBoost is a machine learning algorithm based on gradient boosting decision trees. Different from other gradient boosting algorithms, CatBoost uses a symmetric tree structure, which helps to avoid overfitting and improve reliability (Ding et al., 2021). During the construction of CatBoost trees, each tree is built based on the residuals of the previous tree. This iterative process makes the final prediction more accurate and the model more robust (Zou et al., 2021).

As an ensemble learning algorithm based on classification and regression tree, GBDT consists of Decision Tree and Gradient Boosting. GBDT contains multiple rounds of iterations. The basic classifier generated by each round of iteration is trained on the basis of the residual of the previous round of classifier (residual = true value - predicted value), and then continues to fit the residual of the previous round (Zhang and Jung, 2021).

**Figure 3:**
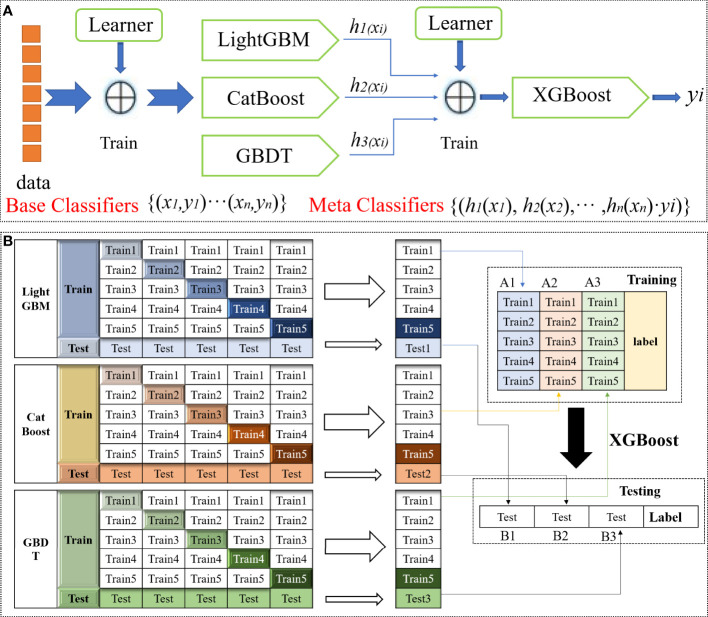
Schematic diagram of the SEL. **(A)** SEL schematic diagram. **(B)** SEL principle flow diagram.

The SEL framework generalizes the output values of multiple models to improve the overall classification performance by using the classification results of the base model as the input data of the meta-model (Fu et al., 2022). The SEL principle was shown in **Figure 3A**. This study stacks four base models (XGBoost, LightGBM, GBDT, CatBoost) to build an ensemble learning model. When using SEL, the original dataset is divided into sub-datasets, which are then used as input data for different base learners in the first layer. The predicted values from the first layer are used as input data for the second layer to train the base learner. The final predicted value comes from the model in the second layer.

As shown in **Figure 3B**. 1)Divide the data into training and test sets. Then divide the training set into five parts (train1, train2, train3, train4, train5). 2)Select the base model. Choose LightGBM, CatBoost and GBDT as base models. For the base model part: use 1 copy as the validation set in turn, and the remaining 4 copies as the training set, perform 5-fold cross-validation for model training, and then make predictions on the test set. This will get 5 predictions trained by the base model on the training set and 1 prediction B1 on the test set. Combine these five vertical overlaps to get A1. 3)After the three base models are trained, the predicted values of the three models on the training set are taken as three features (A1, A2, A3) respectively, and the XGBoost model is used for training to establish the XGBoost model. 4)Using the trained XGBoost model, make predictions on the values of the three features (B1, B2, B3) constructed from the predicted values on the test set before the three base models, and get the final predicted category.

### 2.7 Model evaluation

The hyperspectral data of 70% of the peanut seed samples were selected as training data by random sampling, and the remaining 30% of the data were used as the test set. The machine learning algorithm was used to build a discriminant model to verify the logical properties of the feature response of peanut seeds HSI. Using Modeling Average Time, Accuracy, Log Loss, and Hamming Loss to evaluate the effect of model training predictions (Leng et al., 2020; Huang et al., 2021; Wang et al., 2021c). Log Loss The negative logarithm of the probability that the true probability occurs for a given classifier, conditional on the prediction probability. The smaller the value, it is proved that the probability estimates more accurate, the ideal model. Hamming Loss is used to investigate the misclassification of samples on a single tag, that is, relevant tags do not appear in the predicted tag set or irrelevant tags appear in the predicted tag set. The smaller the index value, the better the model performance. The smaller the value, it is proved that the probability estimates more accurate, the ideal model. Hamming Loss is used to investigate the misclassification of samples on a single tag, that is, relevant tags do not appear in the predicted tag set or irrelevant tags appear in the predicted tag set. The smaller the index value, the better the model performance. The smaller the value, it is proved that the probability estimates more accurate, the ideal model. These evaluation parameters are calculated as follows:


(1.2) $$A\text{ccuracy}=\frac{TP+TN}{TP+FN+FP+TN}$$

In the formula, FP represents the correct sample in the wrong sample, TN represents the wrong sample in the real sample, TP represents the predicted correct sample in the real sample, and FN represents the wrong sample in the incorrect sample.


(1.3) $$Log\;loss=-\frac{1}{N}\sum_{i=1}^{N}\sum_{j=1}^{M}y_{ij}\log(p_{ij})$$

In the formula, N is the number of samples, M is the number of categories, when the ith sample belongs to category j, *Y*_ij_ is 1, otherwise it is 0; *P*_*ij*_ is the probability that the ith sample is predicted to be the jth category.


(1.4) $$\text{Hamming loss=}\frac{1}{N}\sum_{i=1}^{N}\frac{XOR(Y_{ij},P_{\text{ij}})}{L}$$

In the formula, N represents the number of samples, L is the number of label samples, *Y*_ij_ is the actual value of the jth component in the ith predicted value, *P*_*ij*_ is the predicted value of the jth component in the ith predicted value, XOR represents XOR operation.

## 3 Results and discussion

### 3.1 Spectral characteristics of peanut seeds

**Figure 4:**
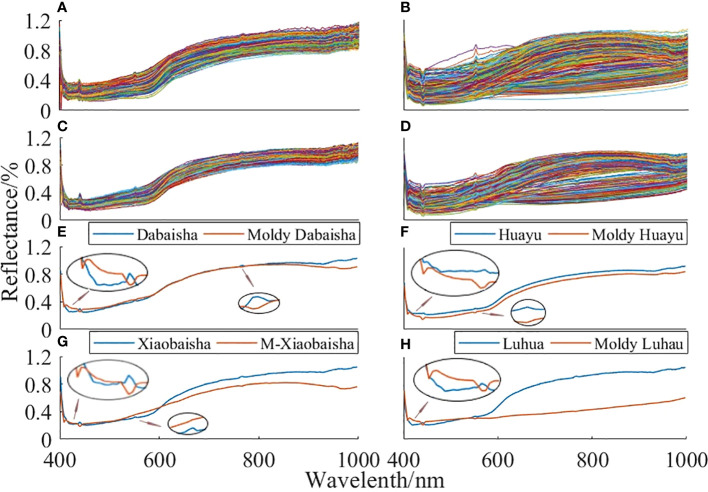
Spectral curves of peanut seeds. **(A)** The original spectral curves of four varieties of peanut seeds; **(B)** the original spectral curves of mildewed peanut seeds; **(C)** the spectral curves after MF pretreatment; **(D)** spectral curves of moldy peanut seeds after MF pretreatment; **(E–H)** are the average spectral curves of four healthy peanut seeds and moldy peanut seeds, respectively.

Black and white correction was performed on the hyperspectral data of the sample, which effectively eliminated the influence of external factors on the spectral data of the sample. **Figures 4A, B** show the raw spectra of peanut seeds and moldy peanut seed samples, and **Figures 4C, D** show the spectra of peanut seeds and moldy peanut seed samples after MF treatment. Compared with the original spectra, the MF-processed spectral data have similar trends to the original spectra. After preprocessing, the noise interference of hyperspectral data is reduced, and the spectral curve is smoother. **Figures 4E–H** show the spectral changes before and after mildewing of four kinds of peanut seeds. Studies have shown that there are obvious spectral differences in the visible light region of 400-450nm, which is related to the color change of peanut seeds after mold (Fernandez-Ibanez et al., 2009; Wang et al., 2015). The reflectance in the visible light region of 400-600nm is generally low, because the pigments such as anthocyanin and chlorophyll in the peanut skin strongly absorb light (He et al., 2021). In the 700 nm-1000 nm spectral band, there are obvious differences in spectral reflectance, mainly caused by the organic chemical bonds of peanut seeds (Kimuli et al., 2018). At small concentrations, its effect on the spectrum may be suppressed by more conspicuous kernel color, so more aflatoxin spectral information is expected in the NIR region between 700 and 1000 nm (Fernandez-Ibanez et al., 2009).

### 3.2 Visualization of hyperspectral data

**Figure 5:**
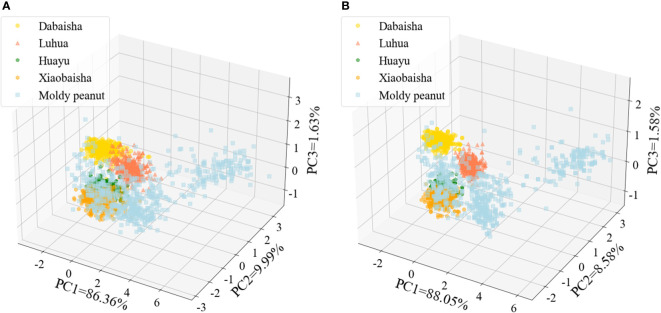
PCA visualization of hyperspectral data of peanut seeds. **(A)** RAW-PCA; **(B)** MF-PCA.

Using PCA algorithm, the first three principal components are selected as data representatives to intuitively reflect the differences between samples. The raw and MF preprocessed hyperspectral data are visualized through PCA algorithm, as shown in **Figures 5A, B**. PC1, PC2 and PC3 are the contribution rates of the first three principal components and also represent the variance of the data they carry. The contribution rate represents the sum of the eigenvalues of each principal component divided by the eigenvalues. In space, PCA can be understood as projecting the original data to a new coordinate system. PC1 respectively represents the change interval of the first new variable obtained by some transformation of multiple variables in the data; PC2 represents the change range of the second new variable obtained by some transformation of multiple variables in the original data; Similarly, PC3 can be obtained. In the raw and MF preprocessed spectral data, the cumulative weights of the first three principal components reached 98.03% and 98.21%, respectively, reflecting the main information of peanut seeds. The first three principal components were selected for principal component analysis of spectral data. Its purpose is to retain the main information of spectral data to the maximum extent, prevent data missing, reduce the redundancy of spectral data, and meet the requirement that the cumulative contribution rate of principal component analysis is greater than 80% (Lee and Jemain, 2021). Spectral data was visualized using PCA, presenting five peanut seed samples as “clusters”. When reduced to three dimensions, it can be seen from **Figures 5A, B** that MF-PCA can better display the five peanut seed samples as “clusters”. RAW-PCA has more intersections and shows poorer results. Each sample in MF-PCA has its own spatial distribution characteristics, which show the best results, which is consistent with the results obtained later with MF as model input. Therefore, by visualizing the preprocessed spectral data through the PCA algorithm, the spatial distribution of the preprocessed spectral data can be clearly seen from the visualized image. After PCA visualization, it can be seen intuitively that five peanut seed samples can be classified. This analysis provides the basis for the following classification model.

### 3.3 Classification model using full spectral

**Figure 6:**
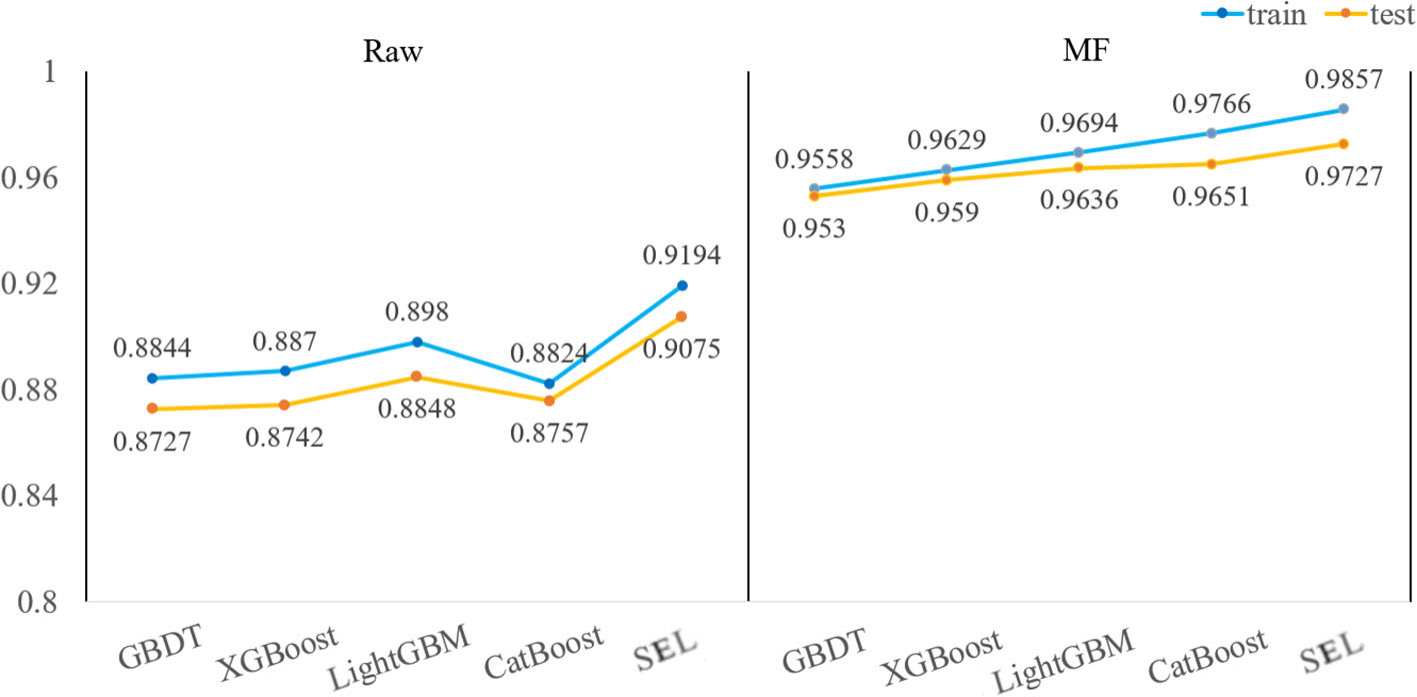
The training set and test set accuracy of the original hyperspectral data and the MF preprocessed hyperspectral data as input for the five models.

The MF spectral preprocessing method combined with five classification models (XGBoost, LightGBM, CatBoost, GBDT, SEL) is used to construct classification models. MF effectively eliminates the noise impact of spectral data, and the classification accuracy has been significantly improved. MF has excellent performance on hyperspectral data. After MF pretreatment, the classification model can accurately identify peanut seed varieties and mildew. **Figure 6** visually shows the accuracy of the test set and training set of each classification model. When using full spectral data as the model input, the accuracy of spectral data is significantly improved after MF preprocessing, among which, the accuracy growth rates of model training set and test set are 6.63-9.42% and 6.52-8.94%. The classification accuracy of MF-SEL is 98.57% and 97.27% on the training and test sets. Moreover, the Log Loss and Hamming Loss have the lowest values of 4906.57 and 0.027273, and the modeling time of MF-SEL is 1.2678s. Comparing the five classification models, the training set and test set accuracy of SEL are higher than the other four classification models. SEL makes the best of the synergistic advantages of different base learners to achieve the effect of complementary integration, thereby improving the accuracy of classification models (Zandi et al., 2022).

### 3.4 Calibrate model using selected spectra

#### 3.4.1 Feature wavelength selection

**Figure 7:**
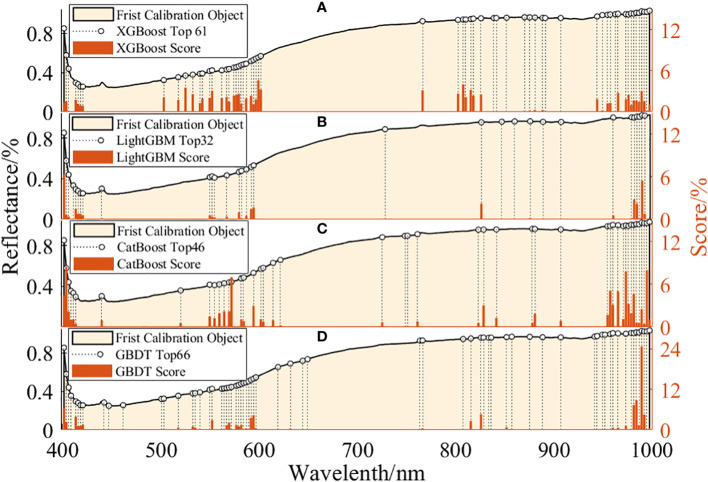
Characteristic wavelength distribution and contribution rate of characteristic wavelengths for four variable selection methods. **(A)** XGBoost; **(B)** LightGBM; **(C)** CatBoost; **(D)** GBDT.

The original spectral data collected by the hyperspectral system consists of a large number of wavebands with multiple collinearity (Khan et al., 2022). XGBoost, LightGBM, CatBoost and GBDT rank the importance of 235 wavelength respectively, and the number of effective wavelengths they select is 61, 32, 46 and 66. **Figure 7** intuitively shows the distribution of characteristic wavelengths selected by different feature selection methods, as well as the importance score of each wavelength. The characteristic wavelengths selected by the four feature selection methods are similar, mainly distributed in 400-450nm, 500-600nm, 790-830nm and 968-1000nm. This also means that there is more differential information near these bands. Selecting some important variables helps to develop a more powerful and concise classification model. Among them, there are obvious spectral differences in the visible light region of 400-450nm, which is related to the color change of peanut seeds after mold (Fernandez-Ibanez et al., 2009; Wang et al., 2015). The reflectance in the visible light region of 400-600nm is generally low, because the pigments such as anthocyanin and chlorophyll in the peanut skin strongly absorb light (He et al., 2021). 800nm is associated with third overtone N-H stretch and third overtone C-H, 968-1000nm is associated with second overtone O-H stretch and second overtone N-H stretch (Kimuli et al., 2018). The experimental results show that the four variable selection methods have similar general trends on the selected characteristic wavelengths. These characteristic wavelengths will be an important basis for finally distinguishing different peanut seed samples. Therefore, the wavelengths selected in the above process were used as input to the subsequent classification model.

#### 3.4.2 Classification model using selected spectra

**Figure 8:**
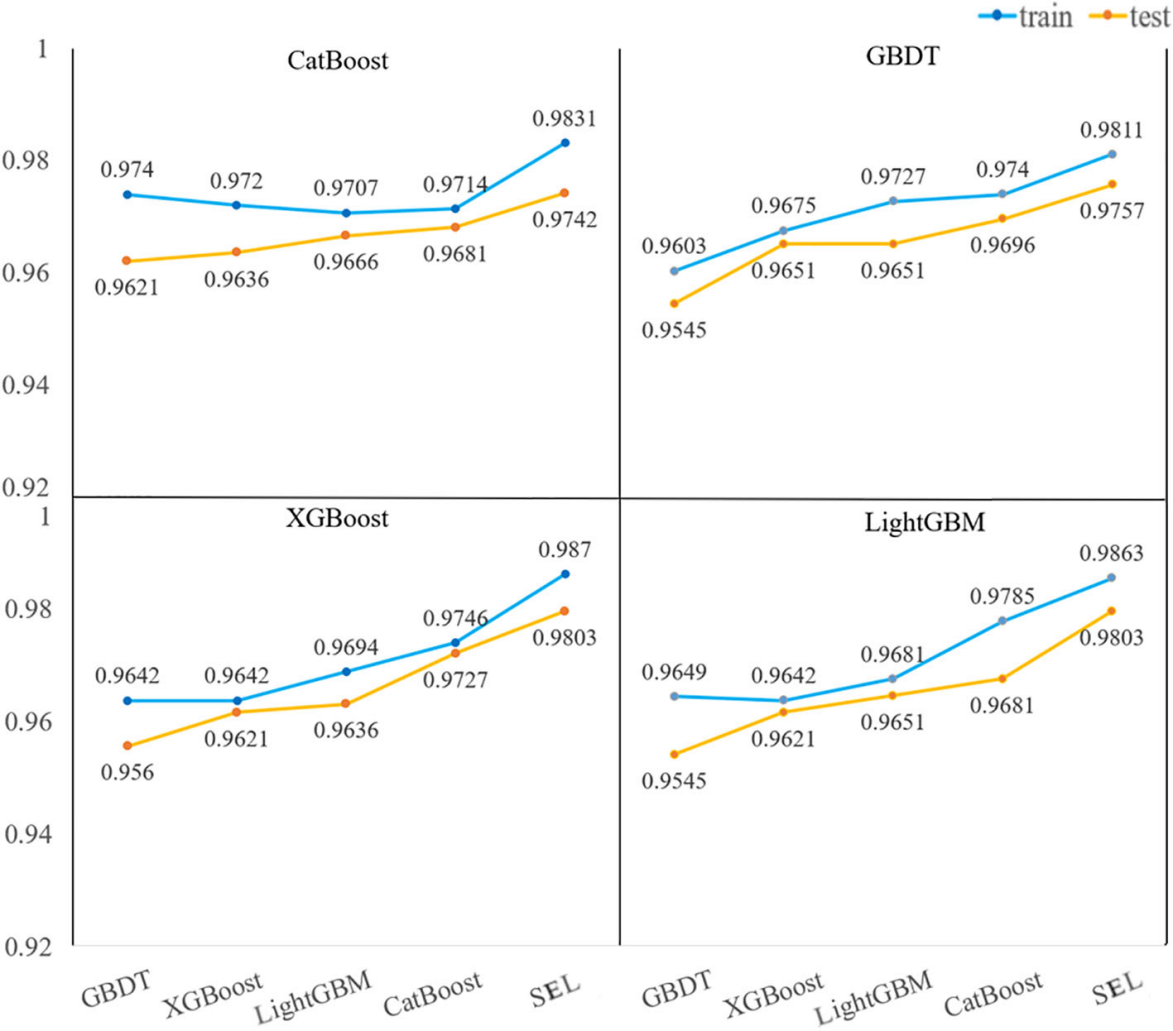
The training and test set accuracies of the feature wavelengths selected by the four variable selection methods as the model input.

The hyperspectral data preprocessed by MF is used to construct classification models through four variable selection methods (XGBoost, LightGBM, CatBoost, GBDT) combined with five classification models (XGBoost, LightGBM, CatBoost, GBDT, SEL). The evaluation indicators of the classification results of each model are summarized in **Table 1**. From the classification results of all classification models in **Table 1**, all classification models can accurately identify the variety of peanut seeds and the mildewed peanut seeds. **Figure 8** visually shows the accuracy of the test set and training set of each classification model. SEL also shows its excellence when using the characteristic wavelengths screened out by different variable selection methods as model inputs. Better classification results were obtained by using fewer wavelengths. Compared with the classification results in **Table 2**, when the characteristic wavelength selected by the variable selection method is used as the model input, the classification accuracy and modeling time are improved. Especially for Stack, the modeling time is improved by about 0.8s. This is due to the fact that the variable selection method selects the more recognizable characteristic wavelengths as the model input, which reduces the redundancy of the data (Chen et al., 2020). Experimental results show that MF-LightGBM-SEL achieves the best classification results. LightGBM selects 17 characteristic wavelengths as model input, and the accuracy rates of training set and test set reach 98.63% and 98.03%, respectively. Log Loss and Hamming Loss are 4733.88 and 0.019697 respectively, and the modeling time is 0.3701s.

**Table 1:** Evaluation indicators for the classification results of peanut seed samples using four variable selection methods combined with five classification models.

Models	Number	Train-accuracy	Test-accuracy	Log loss	Hamming loss	Time(s)
XGBoost -XGBoost	50	96.42%	96.21%	5148.34	0.037879	0.1695
XGBoost -LightGBM	48	96.94%	96.36%	5113.81	0.036364	0.0438
XGBoost -CatBoost	53	97.46%	97.27%	4906.57	0.027273	0.3239
XGBoost -GBDT	47	96.42%	95.60%	5286.51	0.043939	0.1017
XGBoost -SEL	39	98.70%	98.03%	4733.88	0.019697	0.4814
LightGBM -XGBoost	21	96.43%	96.21%	5148.35	0.037879	0.1017
LightGBM -LightGBM	26	96.82%	96.52%	5079.27	0.034848	0.0319
LightGBM -CatBoost	23	97.86%	96.82%	5010.19	0.031818	0.2184
LightGBM -GBDT	32	96.49%	95.45%	5321.04	0.045455	0.0618
LightGBM -SEL	17	98.64%	98.03%	4733.88	0.019697	0.3701
CatBoost -XGBoost	21	97.21%	96.36%	5113.81	0.036364	0.0908
CatBoost -LightGBM	15	97.08%	96.67%	5044.73	0.033333	0.0289
CatBoost -CatBoost	20	97.14%	96.82%	5010.19	0.031818	0.1775
CatBoost -GBDT	20	97.40%	96.21%	5148.35	0.037879	0.0848
CatBoost -SEL	15	98.31%	97.42%	4872.04	0.025758	0.4023
GBDT -XGBoost	29	96.75%	96.52%	5079.27	0.034848	0.1157
GBDT -LightGBM	25	97.27%	96.52%	5079.27	0.034848	0.0389
GBDT -CatBoost	23	97.40%	96.97%	4975.65	0.030303	0.2429
GBDT -GBDT	25	96.04%	95.45%	5321.04	0.045455	0.0539
GBDT -SEL	16	98.12%	97.58%	4837.50	0.024242	0.4249

(Number represents the number of characteristic wavelengths for the optimal result of the model).

**Table 2:** Evaluation indicators of MF combined with five classification models for the classification results of peanut seed samples.

Models	Train-accuracy	Test-accuracy	Log loss	Hamming loss	Time(s)
RAW-XGBoost	88.70%	87.42%	7151.59	0.125758	0.6133
RAW-LightGBM	89.81%	88.48%	6909.82	0.115152	0.1326
RAW-CatBoost	88.24%	87.57%	7117.05	0.124242	0.7431
RAW-GBDT	88.44%	87.27%	7186.13	0.127273	0.3471
RAW-SEL	91.94%	90.75%	6391.74	0.092424	1.3045
MF-XGBoost	96.29%	95.91%	5217.42	0.040909	0.5593
MF-LightGBM	96.94%	96.36%	5113.80	0.036364	0.1316
MF-CatBoost	97.66%	96.51%	5079.27	0.034848	0.9736
MF-GBDT	95.58%	95.30%	5355.58	0.04697	0.3071
MF-SEL	98.57%	97.27%	4906.57	0.027273	1.2678

### 3.5 Classification performance analysis of stacked ensemble models

**Figure 9:**
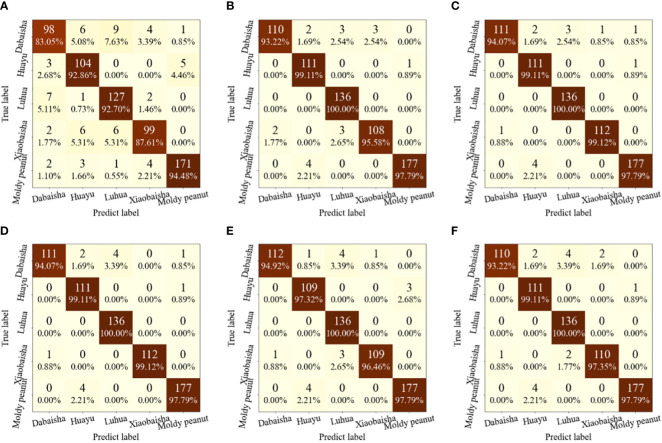
Confusion matrix for the training set of SEL. **(A)** RAW-Stack; **(B)** MF-Stack **(C)** MF-XGBoost-SEL; **(D)** MF-LightGBM-SEL; **(E)** MF-CatBoost-SEL; **(F)** MF-GBDT-SEL.

**Figures 9A–F** show confusion matrices derived from RAW, MF, and hyperspectral data selected by different variable selection methods as SEL input. Except for RAW-SEL, the other SELs achieved 97.79% recognition rate for moldy peanut seed samples. Compared with the RAW-SEL model test set, the accuracy rate is increased by 3.31%, which is due to the good data processing ability of MF that eliminates the noise effect of hyperspectral data. Compared with full-wavelength modeling, the characteristic wavelengths obtained by using the four variable selection methods are also greatly improved. Especially for the peanut seeds of Luhua, the classification accuracy rate reaches 100%. This is due to the use of characteristic wavelength modeling to reduce the problems of collinearity and redundancy in hyperspectral data. Preprocessing the original spectrum and using four variable selection methods to extract characteristic wavelength variables cannot help improve the accuracy and stability of the SEL, but improve the modeling time. To sum up, by comparing the classification results of SEL and the four basic models, SEL shows excellent classification performance. Compared with the basic classification model, it uses less hyperspectral data and obtains better classification results. These are consistent with the findings of several previous studies, e.g. (Zhang et al., 2020) classifies vegetation based on medium resolution spectral imaging technology and SEL, and its accuracy is 5.1-5.2% higher than other single models. (Fu et al., 2022) constructed a model based on multispectral images and SEL, and found that the integrated learning algorithm produced better classification performance than the basic model, with an overall accuracy rate of 1.6-12.7% higher. The results showed that the stacked ensemble model exhibits superior classification performance.

## 4 Conclusion

In this paper, a fast and accurate nondestructive detection method using HSI technology combined with a stacked machine learning model was proposed to classify peanut seed varieties and moldy peanut seeds. The SEL was formed by stacking and integrating XGBoost, LightGBM, CatBoost, and GBDT algorithms. The MF spectral preprocessing method was used to calibrate the model, and it was found that MF preprocessing can reduce the influence of hyperspectral data noise and greatly improve the accuracy of the model. The accuracy rate of the training set increases by 6.53-8.95%. Among the four variable selection methods, LightGBM exhibits the best performance, which effectively eliminates the collinearity and redundancy problems of hyperspectral data. The characteristic wavelengths screened by the four variable selection methods are used as model input, and the growth rate of the training set accuracy is 0.15-0.91%. Compared with the basic model, the training set accuracy rate increases by 0.6%-3.48%. For the hyperspectral data of 17 characteristic wavelengths selected by MF-LightGBM-SEL, the training set and test set accuracy rate reached 98.63% and 98.03%, respectively, and the modeling time was 0.3701s. Log loss and Hamming Loss are namely 5321.04 and 0.045455. The stacking ensemble algorithm exhibits strong classification ability. Compared with the research of Jin et al. (Qi et al., 2019; Sun et al., 2020; Jin et al., 2022), first of all, this paper classifies peanut seed varieties and mildew, identifies two factors that affect peanut yield, and provides a broader reference for improving the quality of peanut seeds. Secondly, the experimental method studied in this paper adds time parameters, Hamming Loss Log Loss and other evaluation indicators, which makes the efficiency and performance of the model more intuitive. Finally, through the strategy of stacking machine learning models, this paper realizes the accurate identification of peanut seed varieties and mildew. The results show that HSI technology has satisfactory potential in identifying peanut seed varieties and discriminating mildewed peanut seeds. In addition, the method of stacking ensemble algorithm combined with HSI technology provides ideas for rapid identification of peanut seed varieties and mildew identification of peanut seeds.

## Data availability statement

The original contributions presented in the study are publicly available. This data can be found here: https://github.com/wuqingsongwj/Peanut-seed.

## Author contributions

Conceptualization, ZYZ and YCW. Data curation, QSW and QLW. Formal analysis, YPZ, JBZ and QFZ. Funding acquisition, LJX, ZYZ and MZ. Methodology, JW and QSW. Project administration, LJX, ZYZ and MZ. Writing—original draft, QSW. Writing—review and editing, ZYZ and QSW. All authors contributed to the article and approved the submitted version.

## Funding

This study was funded by the Sichuan Science and Technology Program (Grant No. 2022NZZJ0034) and Agricultural University Program (Grant No. 2121997858; Grant No. 2221998028).

## Conflict of interest

The authors declare that the research was conducted in the absence of any commercial or financial relationships that could be construed as a potential conflict of interest.

## Publisher’s note

All claims expressed in this article are solely those of the authors and do not necessarily represent those of their affiliated organizations, or those of the publisher, the editors and the reviewers. Any product that may be evaluated in this article, or claim that may be made by its manufacturer, is not guaranteed or endorsed by the publisher.
